# European Management of Glanzmann's Thrombasthenia: A Survey of Current Clinical Practice

**DOI:** 10.1111/hae.70114

**Published:** 2025-09-11

**Authors:** Mathieu Fiore, Andrea Artoni, Robert Klamroth, Mary Mathias, Roger Schutgens, Roseline d'Oiron, EAHAD Glanzmann Thrombasthenia Working Group

**Affiliations:** ^1^ Department of Haematology University Hospital of Bordeaux Pessac France; ^2^ Biology of Cardiovascular Disease Inserm U1034 Pessac France; ^3^ French Reference Centre For Inherited Platelet Disorders University Hospital of Bordeaux Pessac France; ^4^ Angelo Bianchi Bonomi Hemophilia and Thrombosis Center Fondazione IRCCS Ca' Granda Ospedale Maggiore Policlinico Milan Italy; ^5^ Department of Internal Medicine Hemophilia Treatment Center Vivantes Klinikum im Friedrichshain Berlin Germany; ^6^ Haemophilia Comprehensive Care Centre Great Ormond Street Hospital for Children NHS Foundation Trust London UK; ^7^ Department of Benign Hematology Thrombosis and Haemostasis Van Creveldkliniek University Medical Center Utrecht and University Utrecht Utrecht Netherlands; ^8^ Centre De Référence de L'hémophilie Et Des Maladies Hémorragiques Rares Hôpital Bicêtre AP‐HP; and INSERM Hémostase Inflammation Thrombose HITH U1176 Université Paris‐Saclay Le Kremlin‐Bicêtre France

**Keywords:** clinical practice, Glanzmann's disease, Glanzmann's thrombasthenia, real‐world data, survey

## Abstract

**Introduction:**

Glanzmann's thrombasthenia is a rare inherited platelet disorder characterized by a lack of platelet aggregation. Patients tend to be diagnosed in early childhood with treatment strategies involving a multifaceted approach to prevent and manage bleeding episodes. Unfortunately, there is currently no European consensus regarding the management of GT.

**Aim:**

This initiative aimed to gain an understanding of current clinical management of GT across Europe, with the aim of aligning best practice and improving patient outcomes.

**Methods:**

The authors, on behalf of the EAHAD Glanzmann Working Group, administered an online survey of 57 questions to European haematologists currently involved in the management of patients with GT. The survey covered topics related to diagnosis, treatment access and selection, immunization, peri‐operative management and use of second‐line therapies.

**Results:**

Responses reflected physician consensus around some topics, including peri‐operative treatment, use of recombinant factor VIIa, and concerns around antibody development. However, more varied responses were received on topics such as antibody screening (anti‐*αIIbβ3* antibodies screening conducted by ≤53% of respondents in all countries of interest except France), access to HLA‐matched platelet concentrates (none or limited for 55% of respondents) and duration of platelet transfusions for major surgery (13%–31% for 1, 2, 3 and 4 or more days of transfusions).

**Conclusion:**

Establishing comprehensive guidelines to manage GT will enhance patient outcomes by ensuring patients receive high‐quality and effective care as well as standardize care across different healthcare settings.

## 1 Introduction

Glanzmann's thrombasthenia (GT) is an inherited platelet function disorder typically caused by a deficiency or absence of the platelet‐specific *α_IIb_β_3_* integrin [1], the receptor of fibrinogen and required for platelet aggregation on activated platelets, preventing spontaneous and excessive bleeding during vascular injury/trauma or surgery [2]. GT is categorized into three types: <5% of *α_IIb_β_3_* expression (type I), residual (5%–30%) *α_IIb_β_3_* expression (type II) and ≥20% residual expression (variant) [3]. Patients with GT experience significant mucosal bleeding symptoms, including epistaxis, gingival bleeding, heavy menstrual bleeding, as well as easy bruising and purpura [4, 5].

GT is a relatively rare disorder, with a global incidence of 1:1,000,000, leading to challenges in the establishment of guidelines and clinical practice policies [6]. A few recommendations exist, but there is currently no European consensus, and variation in the treatment of GT exists across Europe.

Although the core diagnostic framework for GT—encompassing clinical evaluation, platelet function testing, flow cytometry, and genetic analysis—is broadly recognized, test availability, laboratory expertise and clinical practice may vary across countries [7]. Diagnosis of GT should involve assessment of clinical presentation and laboratory testing [8]. GT can be definitively diagnosed through platelet testing using light transmission aggregometry (LTA), considered the gold‐standard method [8]. Deficiency in platelet surface glycoproteins can be confirmed by flow cytometry, allowing for rapid identification of both deficient and non‐functioning *α_IIb_β_3_*. DNA analysis can identify specific genetic anomalies associated with each case of GT, allowing diagnostic confirmation and maybe genotype‐phenotype correlations in the future as a research question [8]. Laboratory tests should include a full blood count to evaluate platelets, routine coagulation tests, LTA, *α_IIb_β_3_* quantification at the membrane surface and genetic analysis through sequencing of integrin subunit *α_IIb_* (*ITGA2B*) and *β_3_* (*ITGB3*).

With haemostatic control as the main objective, treatment selection is variable and depends on bleeding phenotype and treatment responsiveness. Limited access to treatment may influence management strategy in some settings [9]. While moderate bleeds may be managed with topical or oral antifibrinolytics, severe bleeds may require systemic haemostatic agents. The most common first‐line systemic therapy for GT is platelet transfusions, but these are associated with risks of anti‐human leukocyte antigens (HLA) and/or anti‐*α_IIb_β_3_* immunization and refractoriness [9, 10, 11].

Platelet concentrates are available from multiple or single donors, which can be genotyped, allowing matching of patients and donors and administration of HLA‐matched concentrates [12]. Despite limited supporting evidence, the administration of apheresis platelet concentrates and, if needed, HLA‐matched concentrates, is still preferred [13, 14, 15]. Prophylactic matching is also recommended in some platelet transfusion guidelines [14]. However, HLA‐matched concentrates may not be readily accessible and the presence of rare phenotypes may complicate their use [16]. Immunization against *α_IIb_β_3_* is more common in patients with type I than in those with type II or variant forms [4, 17, 18]. Development of anti‐*α_IIb_β_3_* antibodies may be affected by the type of *ITGA2B* or *ITGB3* pathogenic variants, including null mutations.

Recombinant factor VIIa (rFVIIa) is also approved as a second‐line therapy in GT in case of refractoriness or when platelets are not readily accessible [19]. The recommended dose for treatment of bleeding episodes and prevention of bleeding in patients undergoing surgery/invasive procedures is 90 µg (80–120 µg) rFVIIa per kg bodyweight every 2–3 h, with a minimum of three doses [19]. However, specific indications of rFVIIa may differ amongst countries. Data from the international Glanzmann Thrombasthenia Registry (GTR) show that rFVIIa is frequently used off‐label, regardless of platelet antibodies and/or transfusion refractoriness [1, 9, 14]. In very rare cases, patients with recurrent, life‐threatening haemorrhages refractory to current therapies can be treated with bone marrow transplantation [20].

Iron supplementation is a common therapy to prevent the risk of secondary anaemia associated with recurrent bleeding episodes [21].

On behalf of the European Association for Haemophilia and Allied Disorders (EAHAD) Glanzmann's Thrombasthenia Working Group [22], we used a survey aimed at practicing haematologists to generate a snapshot of GT clinical management across Europe and better understand variation in current practices.

## 2 Materials and Methods

Fifty‐seven survey questions were divided into the following thematic subsections: general information, laboratory testing for diagnosis, administration of platelets, anti‐HLA immunization, anti‐*α_IIb_β_3_* immunization, rFVIIa, and other treatments. The survey questions were developed during multiple rounds of discussion and review amongst the author group.

The survey included single‐choice questions, free‐text boxes and multiple‐choice questions, for which respondents could select multiple answers and thus the number of responses may exceed the number of respondents. All questions were optional. Responses were considered usable if the respondent clicked ‘Submit’ to mark their answers as final, and if the respondent reported their location as within one of the countries of interest.

Data from the survey were pooled to ensure respondent anonymity. The survey was made public via the online survey platform, SurveyMonkey and launched on 23 January 2023, with flyers distributed at the 2023 EAHAD congress (United Kingdom). The survey remained open for completion until 16 June 2023. This analysis presents data collected throughout this period.

## 3 Results

‘*N* = ‘refers to the total number of responses per question, and ‘*n* =’ refers to the number of respondents who provided each answer to a question. Italicized text indicates standardized answers available for selection during the survey.

### 3.1 General Information

Usable responses from 92 respondents practicing in 15 European countries of interest were included. While usable responses were also submitted by respondents in Turkey (*n* = 1) and Sudan (*n* = 1), these countries were considered outside the scope of this initiative.

Forty‐nine percent of the 92 respondents were from France or the United Kingdom (Table 1). Almost all respondents reported some variation of ‘haematology’ when questioned about their *‘speciality/department’*. Respondents most commonly reported managing <5 patients with GT (48%), followed by 5–10 patients (29%), and 10–20 patients (14%). Only 8 respondents follow >20 patients, in Ireland (*n* = 1), Portugal (*n* = 1), Italy (*n* = 3), France (*n* = 1), Spain (*n* = 1) and the Netherlands (*n* = 1). 45% and 40% reported management of adult patients, or both adult and paediatric patients, respectively. Only 15% manage paediatric patients only. Monitoring via follow‐up visits to the specialized centre ≥once a year, was recommended by 87%.

**TABLE 1:** Geographical distribution of usable responses (*N* = 92) from countries of interest (*N* = 15) to a survey on European clinical management of Glanzmann's thrombasthenia.

Country of interest	Number of usable survey responses submitted
Belgium	1
Finland	1
France	26
Germany	8
Hungary	1
Ireland	2
Italy	6
Norway	1
Poland	1
Portugal	1
Slovenia	2
Spain	11
Switzerland	3
The Netherlands	9
United Kingdom	19
**Total**	92

Ninety‐one physicians provided 731 responses regarding tools used to assess GT disease burden. The percentages presented here reflect the proportions of respondents who answered, ‘I know and use this tool’. ISTH/SSC bleeding assessment tool (ISTH‐BAT [23]) was used for bleeding history (82%), Pictorial Blood Loss Assessment Chart (PBAC [24]) for heavy menstrual bleeding (52%) and Euro‐QoL‐5D for quality of life (30%) [25]. However, <40% considered these tools ‘*Very suitable*’ for documenting disease burden.

Most respondents (73%) include their patients in a specific database (*n* = 18) and/or a database common to other congenital bleeding disorders (*n* = 58).

### 3.2 Laboratory Testing for Diagnosis

Almost all respondents use LTA (98%) and flow cytometry (96%). Eighty‐eight percent reported use of *ITGA2B* and *ITGB3* genotyping. Western‐Blot tests were only used by 8%, with many (33%) reporting that they are unavailable in clinical practice. Generally, 61% (*N* = 92) reported access to emergency laboratory tests for diagnosis.

### 3.3 GT Treatment

#### 3.3.1 Platelet Concentrates

**FIGURE 1:**
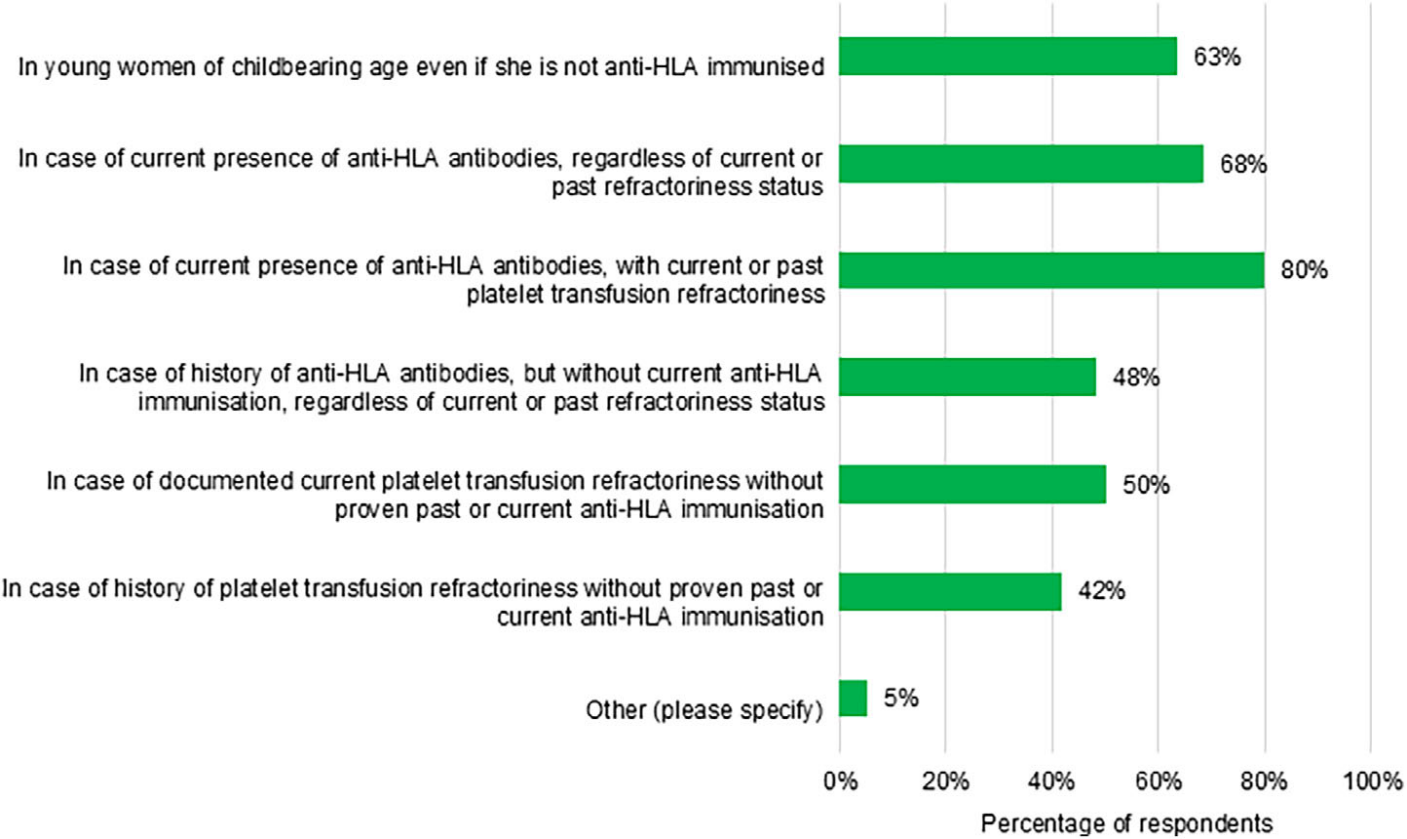
Specific indications in which respondents to a survey of European clinical management of Glanzmann's Thrombasthenia would use HLA‐matched platelet concentrates (*n* = 60).

Ninety‐eight percent (*N* = 91) reported using HLA‐matched platelet concentrates. Thirty‐two percent reported systematic use versus 66% only in specific indications, a breakdown of which is presented in Figure 1.

Most reported limited/no access to HLA‐matched platelets at local blood banks (48% and 7%, respectively), versus 46% (*N* = 92) with unlimited access. Sixty‐one percent reported occasions in clinical practice where use of HLA‐matched platelets was preferred, but they could not wait for/access them.

#### 3.3.2 Anti‐HLA Class‐I Immunization

**FIGURE 2:**
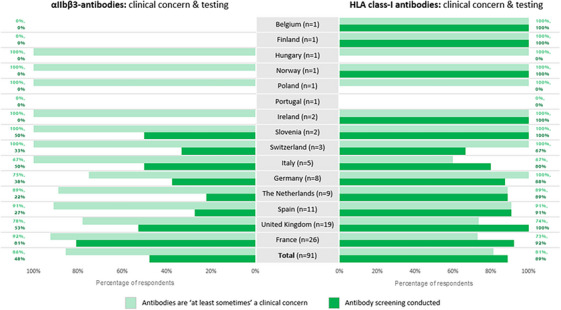
Proportions of respondents that reported anti‐*α_IIb_β_3_* and anti‐HLA class‐I antibodies are ‘at least sometimes’ a clinical concern in the management of GT, and who conducted associated antibody testing (*n* = 91).

Concern around alloimmunization during platelet administration was high, with 82% (*N* = 92) confirming that anti‐HLA class‐I antibodies are ‘at least sometimes’ (‘Always’ + ‘Sometimes’) a clinical concern (Figure 2). Most respondents (89%) monitor anti‐HLA class‐I antibodies, 68% of whom follow‐up on positive results.

Almost all respondents (90%, *N* = 92) perform HLA class‐I phenotyping and/or genotyping: 54% systematically versus 38% occasionally. The purpose of anti‐HLA class‐I antibodies testing and/or HLA class‐I phenotyping varied, including: *‘Prevention of anti‐HLA immunization formation by using HLA‐matched platelet concentrates even if the patient has no history of anti‐HLA antibodies’* (57%); *‘Delivery of HLA‐matched platelet concentrates in cases of anti‐HLA immunization development (with or without platelet transfusion refractoriness)’* (67%); and/or *‘Delivery of HLA‐matched platelet concentrates in case of platelet transfusion refractoriness, but without detected anti‐HLA antibodies’* (33%).

#### 3.3.3 Anti‐*α_IIb_β_3_* Immunization

Eighty‐six percent (*N* = 90) found anti‐*α_IIb_β_3_* antibodies ‘at least sometimes’ a clinical concern. However, only 48% conduct regular screening. National‐level responses are shown in Figure 2. France was the only country in which a large majority reported regular screening (81% vs. ≤53%).

**FIGURE 3:**
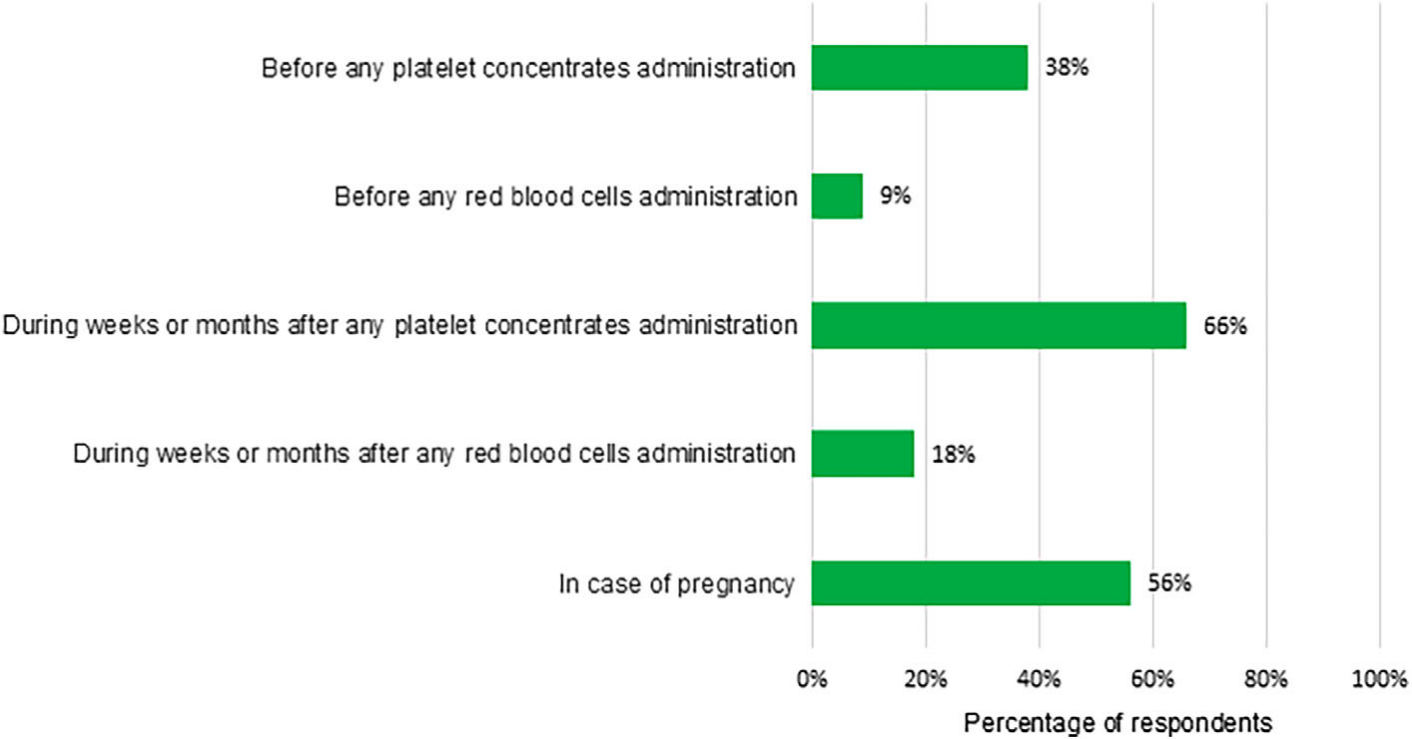
Situations in which respondents would consider anti‐αIIbβ3 antibody screening (*n* = 68).

**FIGURE 4:**
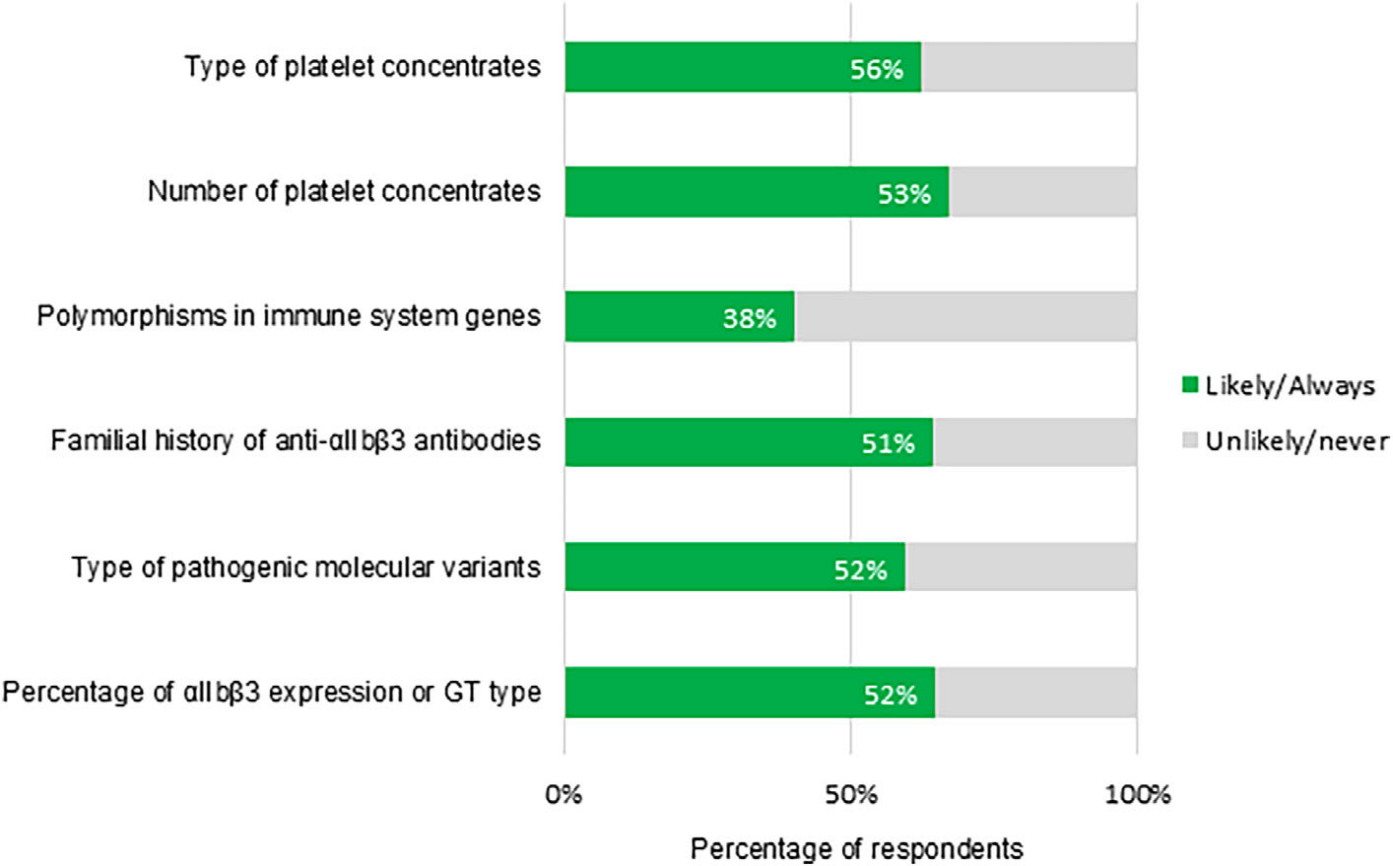
Risk factors for anti‐αIIbβ3 antibodies that would influence clinical strategy (*n* = 84).

Responses regarding scenarios for anti‐*α_IIb_β_3_* antibody screening are presented in Figure 3. Responses (grouped into ‘Likely’ + ‘Always’ and ‘Never’ + ‘Unlikely’) regarding risk factors for anti‐*α_IIb_β_3_* antibodies are summarized in Figure 4.

For detection of *α_IIb_β_3_*‐antibodies, respondents preferred indirect MAIPA (58%), followed by ELISA (19%), flow cytometry (10%) and Luminex technology (10%).

#### 3.3.4 Platelet Transfusion Recovery

One hundred and twenty‐seven responses (*N* = 91) addressed definitions of platelet transfusion inefficiency. Most respondents selected *‘Clinical inefficacy despite sufficient volume of platelets’* (79%) and/or *‘Insufficient platelet count increments’* (45%). Thirteen percent defined platelet transfusion inefficiency as *‘Insufficient proportion of α_IIb_β_3_ positive platelets after transfusion by flow cytometry’*.

Fifty‐five percent (*N* = 92) reported evaluation of platelet transfusion recovery with laboratory testing (national level responses included in supplementary information, Figure S1). Most reported using platelet counts (96%, *N* = 52). Other tests included *‘Flow cytometry’* (percentage evaluation of CD41‐ or CD61 positive transfused platelets) (29%), *‘Platelet Function Analyzer’* (29%), ‘*Platelet aggregation testing’* (17%), *‘Flow cytometry (platelet function testing using PAC‐1 or other markers)’* (13%), and *‘Thromboelastography’* (12%).

#### 3.3.5 rFVIIa

Seventy‐nine percent (*N* = 92) often/always consider using rFVIIa despite lack of antiplatelet immunization or presence of platelet transfusion inefficacy (off‐label). Only 2% never consider using rFVIIa. Qualitative responses suggested off‐label use of rFVIIa to reduce alloimmunization risk in women, with consideration of pregnancies, and/or as treatment or prophylactic cover during surgeries.

Bleeding site and/or severity is considered by most respondents (76% of 90) when selecting rFVIIa. Age and/or sex were considered by 67% (*N* = 89). Sixty‐nine percent (*N* = 89) reported using rFVIIa concomitantly or sequentially with platelet transfusions.

Eighty‐seven percent (*N* = 90) use the recommended dose during surgery/invasive procedures. Possible dose adjustment may be influenced by age (26%), location of bleed/type of surgery (74%), efficacy of treatment during previous episode (71%) or current episode (89%), and thromboembolic risk factors (48%).

#### 3.3.6 Other Treatments

Only 1% (*N* = 91) versus 71% often reported versus never using desmopressin. Chronic oral supplementation was prescribed by most (74%, *N* = 92). Intravenously administered iron for some patients was reported by 77% (*N* = 92), citing *‘Inefficiency of oral administration’* (28%), *‘Chronic and persistent anaemia’* (28%), *‘Digestive intolerance of oral iron’* (26%), and ‘*Acute anaemia’* (19%).

Eighty‐nine percent (*N* = 91) reported that no patients had undergone bone marrow transplantation.

### 3.4 Surgical Management in Adult Patients

#### 3.4.1 Minor Surgery

Seventy‐nine percent (*N* = 80) reported transfusing only one concentrate of platelets pre‐operatively. Eighty‐nine percent (*N* = 85) do not perform second systematic transfusions post‐operatively.

When considering the use of rFVIIa in cases of favourable response, most (56%) proposed >1 day of coverage.

#### 3.4.2 Major Surgery

**FIGURE 5:**
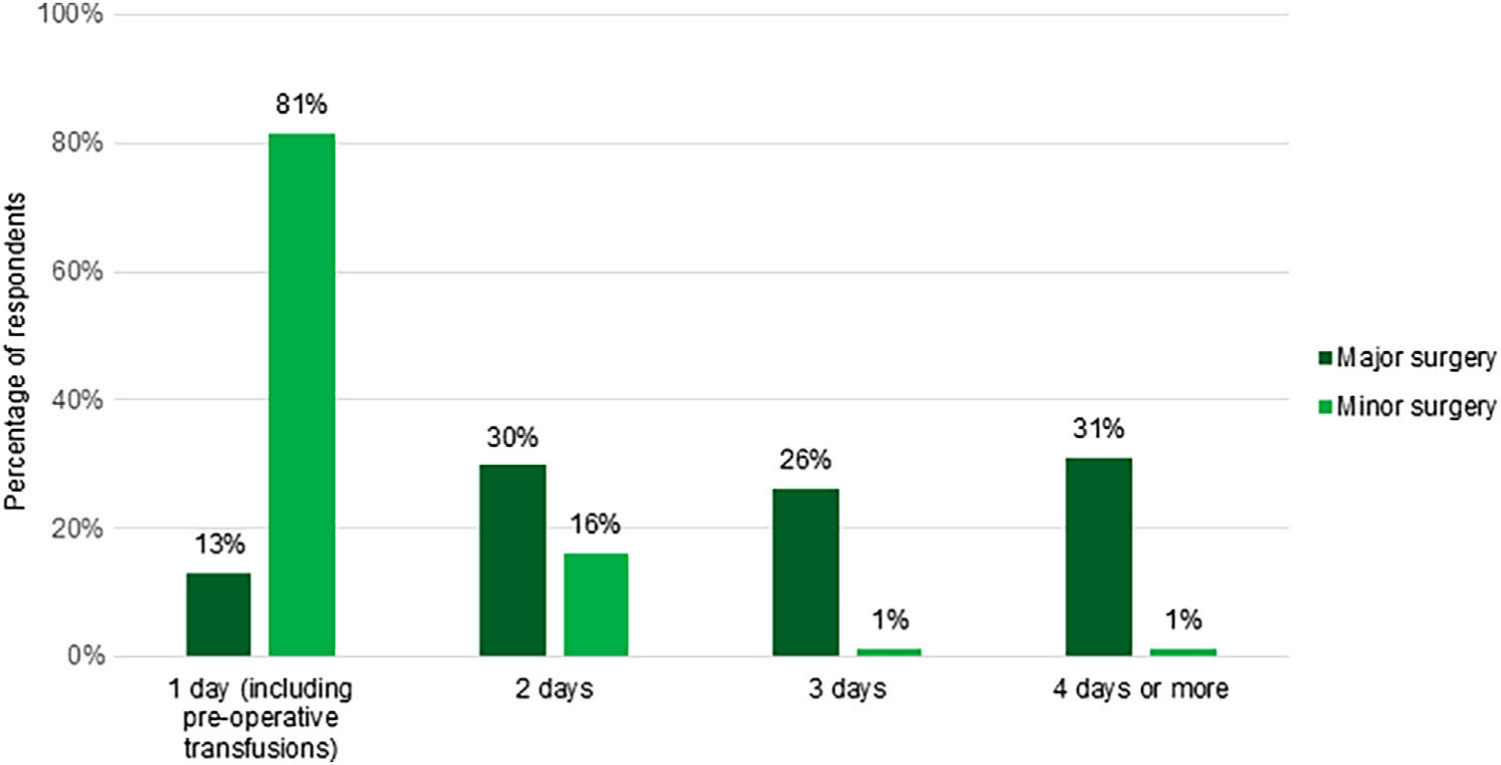
Days of platelet transfusions in cases of major and minor surgery (*n* = 84).

In contrast to minor surgery, most (72%) reported ≥2 concentrates of platelets pre‐operatively. Responses varied regarding second transfusions post‐operatively: 10% and 37% reported systematic transfusions 12 h and 24 h after the first transfusion, respectively. Twenty‐three percent perform second transfusions based on transfusion recovery, monitored through laboratory testing. Days of platelet transfusions during surgery are presented in Figure 5.

**FIGURE 6:**
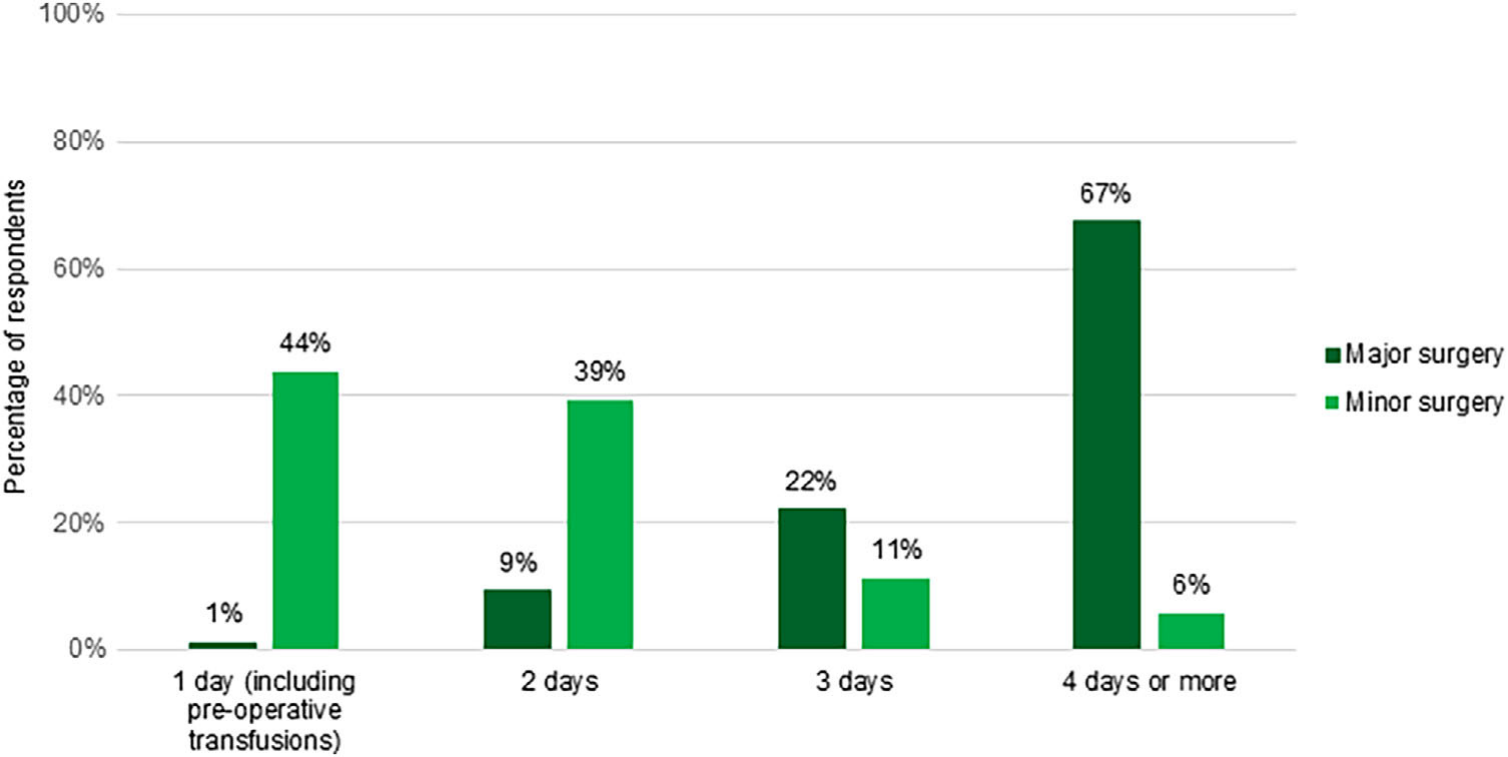
Duration of proposed rFVIIa coverage in cases of minor surgery (*n* = 89) and major surgery (*n* = 86).

Most respondents reported ≥4 days of rFVIIa coverage post‐operatively for major surgery (67%, *N* = 86), versus 1 day for minor surgery (44%, *N* = 89) (Figure 6).

#### 3.4.3 Other Haemostatic Cover

Ninety‐eight percent (*N* = 91) reported use of tranexamic acid during surgery (2% only during minor surgery). Most (62%, *N* = 91) do not use pharmacological thromboprophylaxis during surgery.

## 4 Discussion

Diagnosis and management of GT in clinical practice can be challenging, as it relies on careful analysis of medical history and presentation [8, 26]. Management approaches are often heterogeneous, reflecting variation in treatment centre experience, available treatments and patient‐specific factors. This initiative investigated current European clinical management, collecting survey data from 92 haematologists from 15 countries. During this study, we identified several common viewpoints, while others were less consensual (degree of consensus assessed and agreed by the author group, summarized in Table 2). This diversity in responses highlights the need for greater alignment around best practices, which has the potential to improve patient outcomes.

**TABLE 2:** Overview of consensual and non‐consensual responses to a survey on European clinical management of Glanzmann's thrombasthenia.

High consensus	Low consensus
**General information and laboratory testing**	
Inclusion of patients in national registries Follow‐up of patients Laboratory diagnosis	Use of disease burden measures and assessment of suitability Access to emergency laboratory tests
**Administration of platelet concentrates, including during surgery**	
Overall use of HLA‐matched platelets Definition of transfusion inefficacy Only 1 concentrate of platelets pre‐operatively in cases of minor surgery ≥2 concentrates of platelets pre‐operatively to adult patients undergoing major surgery and more than one day post‐operatively	Use of HLA‐matched platelets either systematically versus in specific indications, or which indications Access to HLA‐matched platelet concentrates Evaluation of platelet transfusion recovery with laboratory testing Days of coverage after major surgery
**Anti‐HLA class‐I and anti‐*αIIbβ3* antibodies**	
Clinical concern surrounding anti‐HLA and anti‐*α_IIb_β_3_* antibodies Monitoring of anti‐HLA class‐I antibodies HLA class‐I phenotyping and/or genotyping	Monitoring of anti‐*α_IIb_β_3_* antibodies Risk factors associated with anti‐*α_IIb_β_3_* antibodies
**rFVIIa**	
Consideration of using rFVIIa despite lack of antiplatelet immunization or presence of platelet transfusion inefficacy (off‐label) Use of the recommended dose Dose adjustment Location of bleed, type of surgery and efficacy of treatment ≥3 days of coverage after major surgery	Indications of off‐label use Days of coverage after minor surgery
**Other therapies**	
Frequent use of tranexamic acid in surgery Use of iron supplementation Rare use of bone marrow transplantation Minimal use of desmopressin	

Abbreviations: HLA, human leukocyte antigens; rFVIIa, recombinant factor VIIa.

### 4.1 Consensual Approaches to Management

National registries/databases allow investigation of patient characteristics, risk factors for disease burden, and different therapies. Our results demonstrate that inclusion of patients in national databases is common. Coordinating databases at scale would enhance visibility of GT management. The EAHAD Glanzmann's Thrombasthenia Working Group recently launched the Glanzmann National History Study (GNHS), which aims to estimate haemorrhagic burden in participants with GT and establish a registry as a potential source for recruitment to research.

These results also showed considerable consensus across European clinical practice regarding diagnostic tools. LTA, flow cytometry and genetic testing remain popular amongst physicians.

For platelet transfusions, guidelines advise obtaining the corrected count increment as a measure of effect/refractoriness in patients with thrombocytopenia [15]. Since patients with GT may have normal or subnormal platelet counts, evaluation of platelet refractoriness was investigated in this survey. Most respondents recognized that the definition varies, depending on clinical progression as patients receive sufficient volume of platelets, confirming that correct evaluation of platelet transfusion efficacy is complicated in GT by normal or sub‐normal platelet counts.

During minor surgery, physicians agree to only transfuse one platelet concentrate pre‐operatively, versus a preference for two platelet concentrates in cases of major surgery. This observation is evident, considering that transfused platelets will compete with patients’ own defective platelets, necessitating a higher volume of platelets at the site of wound healing in cases of major surgery. In the Surgery in Platelet Disorders and Therapeutic Approach (SPATA) study, Orsini et al. evaluated bleeding complications during surgery and preventative measures adopted in 182 procedures involving 89 patients. Findings indicated that inadequate transfusion of platelets correlated with a higher incidence of excessive bleeding [27].

Unfortunately, immune responses against the HLA class‐I system and/or the *α_IIb_β_3_* integrin are elicited in response to platelet transfusions, which should generate great clinical concern [28]. Indeed, >80% of respondents in this study reported that these antibodies are ‘at least sometimes’ a clinical concern, leading to high rates of anti‐HLA class‐I antibody monitoring (89%) and HLA class‐I phenotyping and/or genotyping (90%).

Our study showed that rFVIIa is generally administered with the recommended dose, as previously reported in the GTR [7]. Seventy‐nine percent consider using rFVIIa despite a lack of antiplatelet immunization or presence of platelet transfusion inefficacy, confirming extensive off‐label use. Indeed, GTR data showed that rFVIIa was frequently used in non‐surgical and surgical bleeds (516/829 bleeds and 89/206 procedures), in patients with no confirmed history of antiplatelet antibodies and/or refractoriness; although it should be determined whether this therapeutic option concerned all patients or only those with specific factors. Regarding duration, most respondents (>80%) reported using rFVIIa for ≥3 days post‐operatively in cases of major surgery. Duration for major surgery treated with rFVIIa (alone or in combination with other haemostatic agents) averaged slightly <3 days in the GTR registry, with no reports of post‐surgical bleeding, suggesting that a duration of three days is considered optimal.

Finally, our survey confirmed that patients with GT are considered at increased risk of developing iron deficiency, which is of clinical concern due to implications of reduced stature, reduced growth velocity, altered immune function, and impaired cognitive function in the paediatric population.

### 4.2 Non‐Consensual Management and Unmet Needs

Responses to this survey suggest several non‐consensual management practices. It is perhaps unsurprising that different approaches exist across the haematology community. Delays in diagnosis often occur if patients are managed by general paediatricians or family doctors, who may lack familiarity with GT symptoms and diagnostic criteria. The scarcity of GT means that even institutions with specialized bleeding disorder services may encounter small numbers of affected patients.

While most respondents reported using ISTH‐BAT [23], PBAC [24], and Euro‐QoL‐5D [25] to assess disease burden, some respondents also reported finding these tools unsuitable, signifying the need for tools tailored to GT's unique pathophysiology.

While consensus was reached on laboratory tests for diagnosis, 33%, 3% and 5% of respondents reported that western blots, flow cytometry and genetic testing, respectively, were unavailable in their practice, which impacts diagnosis in emergency settings. This may reflect the scarcity of GT and highlights the need to improve accessibility to reliable laboratory tests to provide rapid assessment during emergencies.

Many respondents reported limited/no access to HLA‐matched platelets from their local blood bank, potentially due to the limited donor pool (particularly in smaller centres), time constraints during matching, and maintaining a sufficient supply of HLA‐matched platelets, which can be logistically challenging. Alternative treatment strategies, like platelet mimic particles or in vitro platelet production, may be valuable in the future.

The likelihood of developing HLA antibodies is higher in patients with GT than in healthy individuals, due to frequent platelet transfusions. Thirty‐two percent use HLA‐matched platelet concentrates systematically, which may be considered high in some countries and low in those that advocate for preventative HLA‐matching, for example, the Netherlands. The question did not specify whether management differs between acute on‐demand treatment of a bleed compared with planned surgery, which may have influenced the result. This element of management would benefit from further investigation.

Only 48% regularly screen for anti‐*α_IIb_β_3_* antibodies, despite most respondents reporting that they are ‘at least sometimes’ a clinical concern. This may be due to challenging laboratory testing (e.g., requirement for specialized assays) compared to more widespread anti‐HLA antibody screening. There appears to be uncertainty around risk factors associated with anti‐*α_IIb_β_3_* antibody development, as consensus was reached on only ≤56% of each specified risk factor.

Sixty‐two percent do not use pharmacological thromboprophylaxis during surgery. However, interpretation of this result is constrained by the question's phrasing, as the nature of surgery was undefined. This result may be compared with the SPATA‐DVT study, where investigators evaluated 210 surgical procedures (155 patients) with well‐defined forms of inherited platelet disorders [29]. No patients with GT in this cohort (*n* = 5) received pharmacologic thromboprophylaxis. The use of low‐molecular‐weight‐heparin was low (10.5%), and two thromboembolic events were registered, both occurring after high‐risk procedures in patients who did not receive thromboprophylaxis (4.7%).

### 4.3 Limitations

This survey study was limited by the low response rates in some countries, with most responses received from France and the United Kingdom, creating a potential bias. The authors agree that a national‐level analysis would provide useful context to these results, but this was agreed to be beyond the scope of this study.

These results provide an overview of management, but there is scope for additional data collection on specific topics, for example, GT in women and girls, menstruation, obstetrics and gynaecology, mild phenotypes, major surgery, perceptions of novel therapies like gene therapy, and cardiovascular risks.

In some cases, the phrasing of the question limited further interpretation of the associated responses. For example, *‘Do you follow‐up anti‐HLA antibodies if they are positive?’* only allows for closed responses and does not elucidate which steps are taken during follow‐up. Additionally, the survey did not specify whether respondents should consider their own practice or the average practice at their centre.

## 5 Conclusions

This survey study demonstrates heterogeneity in several areas of clinical management of GT across Europe. Continued research is essential to refine existing strategies and ensure implementation of best practices. Establishing comprehensive guidelines would enhance patient outcomes by ensuring high‐quality and effective care, standardized across different healthcare settings. Educational programs and consensus statements could be valuable to the treating community.

## Author Contributions

All authors contributed to the conception and design of the study, performance of the analysis, interpretation of the results and the writing of this manuscript. All authors approved the final article.

## Ethics Statement

Ethical approval was not required for this study. Responses to the survey were anonymized, and data were only collected for the purpose of these analyses. No patients were included in this study.

## Conflicts of Interest

M.F. received speaker's fees and/or research grants from Novo Nordisk. A.A. received consultant honoraria from Sanofi. R.K. received consultant fees, speaker's fees and/or research grants from Bayer, Biotest, BioMarin, CSL Behring, Grifols, Kedrion, LFB, Novo Nordisk, Octapharma, Pfizer, Roche/Chugai, Sanofi, SOBI, and Takeda. M.M. received consulting fees from Sobi, payments and support for attending meetings and/or travel from Octapharma and Novo Nordisk, participated on a Data Safety Monitoring Board/advisory board for Sobi, is a secretary of the UKHCDO, and is or has been involved in research for Roche, Sanofi, Novo Nordisk, and Octapharma. R.S. received speaker's fees and/or research grants from Bayer, CSL Behring, Hemab, Novartis, Novo Nordisk, Octapharma, Roche, Sobi, and Takeda. Roger Schutgens also received concizumab from Novo Nordisk and HMB001 from Hemab for in vitro research purposes. R.d'O. has received speaker's fees from Takeda, BioMarin, CSL Behring, LFB, Novo Nordisk, Octapharma, Roche/Chugai, Sobi/Sanofi, uniQure and Spark.

## Supporting information




**Supplement**: European Management of Glanzmann's Thrombasthenia: A Survey of Current Clinical Practice

## Data Availability

The respondent‐level analysis data sets for the research presented are available from the corresponding author on reasonable request.

## References

[1] J. P. Botero , K. Lee , B. R. Branchford , et al., “Glanzmann Thrombasthenia: Genetic Basis and Clinical Correlates,” Haematologica 105, no. 4 (2020): 888–894. 32139434 10.3324/haematol.2018.214239 PMC7109743

[2] P. K. Chaudhary , S. Kim , and S. Kim , “An Insight Into Recent Advances on Platelet Function in Health and Disease,” International Journal of Molecular Sciences 23, no. 11 (2022): 6022. 35682700 10.3390/ijms23116022 PMC9181192

[3] M. Fiore , R. d'Oiron , X. Pillois , and M. C. Alessi , “Anti‐AlphaIIb Beta3 Immunization in Glanzmann Thrombasthenia: Review of Literature and Treatment Recommendations,” British Journal of Haematology 181, no. 2 (2018): 173–182. 29611179 10.1111/bjh.15087

[4] S. Bellucci and J. Caen , “Molecular Basis of Glanzmann's Thrombasthenia and Current Strategies in Treatment,” Blood Reviews 16, no. 3 (2002): 193–202. 12163005 10.1016/s0268-960x(02)00030-9

[5] A. T. Nurden , “Glanzmann Thrombasthenia,” Orphanet Journal of Rare Diseases 1 (2006): 10. 16722529 10.1186/1750-1172-1-10 PMC1475837

[6] I. Iqbal , S. Farhan , and N. Ahmed , “Glanzmann Thrombasthenia: A Clinicopathological Profile,” Journal of the College of Physicians and Surgeons–Pakistan 26, no. 8 (2016): 647–650. 27539755

[7] M. C. Poon , R. d'Oiron , R. B. Zotz , et al., “The International, Prospective Glanzmann Thrombasthenia Registry: Treatment and Outcomes in Surgical Intervention,” Haematologica 100, no. 8 (2015): 1038–1044. 26001792 10.3324/haematol.2014.121384 PMC5004419

[8] T. Solh , A. Botsford , and M. Solh , “Glanzmann's Thrombasthenia: Pathogenesis, Diagnosis, and Current and Emerging Treatment Options,” Journal of Blood Medicine 6 (2015): 219–227. 26185478 10.2147/JBM.S71319 PMC4501245

[9] M. C. Poon , G. Di Minno , R. d'Oiron , and R. Zotz , “New Insights into the Treatment of Glanzmann Thrombasthenia,” Transfusion Medicine Reviews 30, no. 2 (2016): 92–99. 26968829 10.1016/j.tmrv.2016.01.001

[10] M. C. Poon , R. D'Oiron , M. Von Depka , K. Khair , C. Negrier , and A. Karafoulidou , “Prophylactic and Therapeutic Recombinant Factor VIIa Administration to Patients With Glanzmann's Thrombasthenia: Results of an International Survey,” Journal of Thrombosis and Haemostasis 2, no. 7 (2004): 1096–1103. 15219192 10.1111/j.1538-7836.2004.00767.x

[11] R. Conte , D. Cirillo , F. Ricci , C. Tassi , and P. L. Tazzari , “Platelet Transfusion in a Patient Affected by Glanzmann's Thrombasthenia With Antibodies Against GPIIb‐IIIa,” Haematologica 82, no. 1 (1997): 73–74. 9107089

[12] A. L. Kreuger , G. W. Haasnoot , J. A. E. Somers , et al., “Ensuring HLA‐Matched Platelet Support Requires an Ethnic Diverse Donor Population,” Transfusion 60, no. 5 (2020): 940–946. 32086954 10.1111/trf.15728 PMC7317777

[13] J.‐A. S. Bell and G. F. Savidge , “Glanzmann's Thrombasthenia Proposed Optimal Management During Surgery and Delivery,” Clinical and Applied Thrombosis/Hemostasis 9 (2003): 167–170. 12812388 10.1177/107602960300900213

[14] L. J. Estcourt , J. Birchall , S. Allard , et al., “Guidelines for the Use of Platelet Transfusions,” British Journal of Haematology 176, no. 3 (2017): 365–394. 28009056 10.1111/bjh.14423

[15] D. L. Skerrett , New York State Council on Human Blood and Transfusion Services: Guidelines for the Administration of Platelets, 3rd ed. (New York State Council on Human Blood and Transfusion Services, 2012).

[16] C. S. Weinstock and M. Schnaidt , “Human Leucocyte Antigen Sensitisation and Its Impact on Transfusion Practice,” Transfusion Medicine and Hemotherapy 46, no. 5 (2019): 356–369. 31832061 10.1159/000502158 PMC6876597

[17] A. T. Nurden , X. Pillois , and D. A. Wilcox , “Glanzmann Thrombasthenia: State of the Art and Future Directions,” Seminars in Thrombosis and Hemostasis 39, no. 6 (2013): 642–655. 23929305 10.1055/s-0033-1353393 PMC4011384

[18] A. T. Nurden and P. Nurden , “Inherited Disorders of Platelet Function: Selected Updates,” Journal of Thrombosis and Haemostasis 13, no. Suppl 1 (2015): S2–S9. 26149024 10.1111/jth.12898

[19] M.‐C. Poon , “The Use of Recombinant Activated Factor VII in Patients With Glanzmann's Thrombasthenia,” Thrombosis and Haemostasis 121, no. 3 (2021): 332–340. 33124022 10.1055/s-0040-1718373 PMC7895543

[20] V. Wiegering , K. Sauer , B. Winkler , M. Eyrich , and P. G. Schlegel , “Indication for Allogeneic Stem Cell Transplantation in Glanzmann's Thrombasthenia,” Hamostaseologie 33, no. 4 (2013): 305–312. 23868573 10.5482/HAMO-12-08-0014

[21] A. Lee , C. L. Maier , and G. Batsuli , “Iron Deficiency Anemia and Bleeding Management in Pediatric Patients With Bernard‐Soulier Syndrome and Glanzmann Thrombasthenia: A Single‐Institution Analysis,” Haemophilia 28, no. 4 (2022): 633–641. 35412688 10.1111/hae.14559 PMC9810257

[22] “EAHAD Glanzmann's Thrombasthenia Working Group,” EAHAD, https://www.eahad.org/about‐eahad/glanzmann‐thrombasthenia‐working‐group/.

[23] F. Rodeghiero , A. Tosetto , T. Abshire , et al., “ISTH/SSC Bleeding Assessment Tool: A Standardized Questionnaire and a Proposal for a New Bleeding Score for Inherited Bleeding Disorders,” Journal of Thrombosis and Haemostasis 8, no. 9 (2010): 2063–2065. 20626619 10.1111/j.1538-7836.2010.03975.x

[24] J. M. Higham , R. W. O'Brien , and R. W. Shaw , “Assessment of Menstrual Blood Loss Using a Pictorial Chart,” British Journal of Obstetrics and Gynaecology 97, no. 8 (1990): 734–739. 2400752 10.1111/j.1471-0528.1990.tb16249.x

[25] R. Rabin and F. de Charro , “EQ‐5D: A Measure of Health Status From the EuroQol Group,” Annals of Medicine 33, no. 5 (2001): 337–343. 11491192 10.3109/07853890109002087

[26] G. Di Minno , R. B. Zotz , R. d'Oiron , et al., “The International, Prospective Glanzmann Thrombasthenia Registry: Treatment Modalities and Outcomes of Non‐Surgical Bleeding Episodes in Patients With Glanzmann Thrombasthenia,” Haematologica 100, no. 8 (2015): 1031–1037. 26001793 10.3324/haematol.2014.121475 PMC5004418

[27] S. Orsini , P. Noris , L. Bury , et al., “Bleeding Risk of Surgery and Its Prevention in Patients With Inherited Platelet Disorders,” Haematologica 102, no. 7 (2017): 1192–1203. 28385783 10.3324/haematol.2016.160754 PMC5566025

[28] E. J. Huisman , N. Holle , M. Schipperus , et al., “Should HLA and HPA‐matched Platelet Transfusions for Patients With Glanzmann Thrombasthenia or Bernard‐Soulier syndrome be Standardized Care? A Dutch Survey and Recommendations,” Transfusion 64, no. 5 (2024): 824–838. 38642032 10.1111/trf.17824

[29] F. Paciullo , L. Bury , P. Noris , et al., “Antithrombotic Prophylaxis for Surgery‐Associated Venous Thromboembolism Risk in Patients With Inherited Platelet Disorders. The SPATA‐DVT Study,” Haematologica 105, no. 7 (2020): 1948–1956. 31558677 10.3324/haematol.2019.227876 PMC7327644

