# Supplementary material for: European Management of Glanzmann's Thrombasthenia: A Survey of Current Clinical Practice
Source: Haemophilia. 2025 Sep 11;31(6):1261–70. doi: 10.1111/hae.70114 (PMC12612366; doi:10.1111/hae.70114)
Supplement: Supplementary file 1 — Supplement: European Management of Glanzmann's Thrombasthenia: A Survey of Current Clinical Practice [file HAE-31-1261-s001.pdf]

Supplement

European Management of Glanzmann's Thrombasthenia:

A Survey of Current Clinical Practice

1. Additional results

Distribution of emergency card

- A large majority (86%) of respondents do issue emergency cards to their patients.

Use of laboratory testing to evaluate platelet transfusion recovery

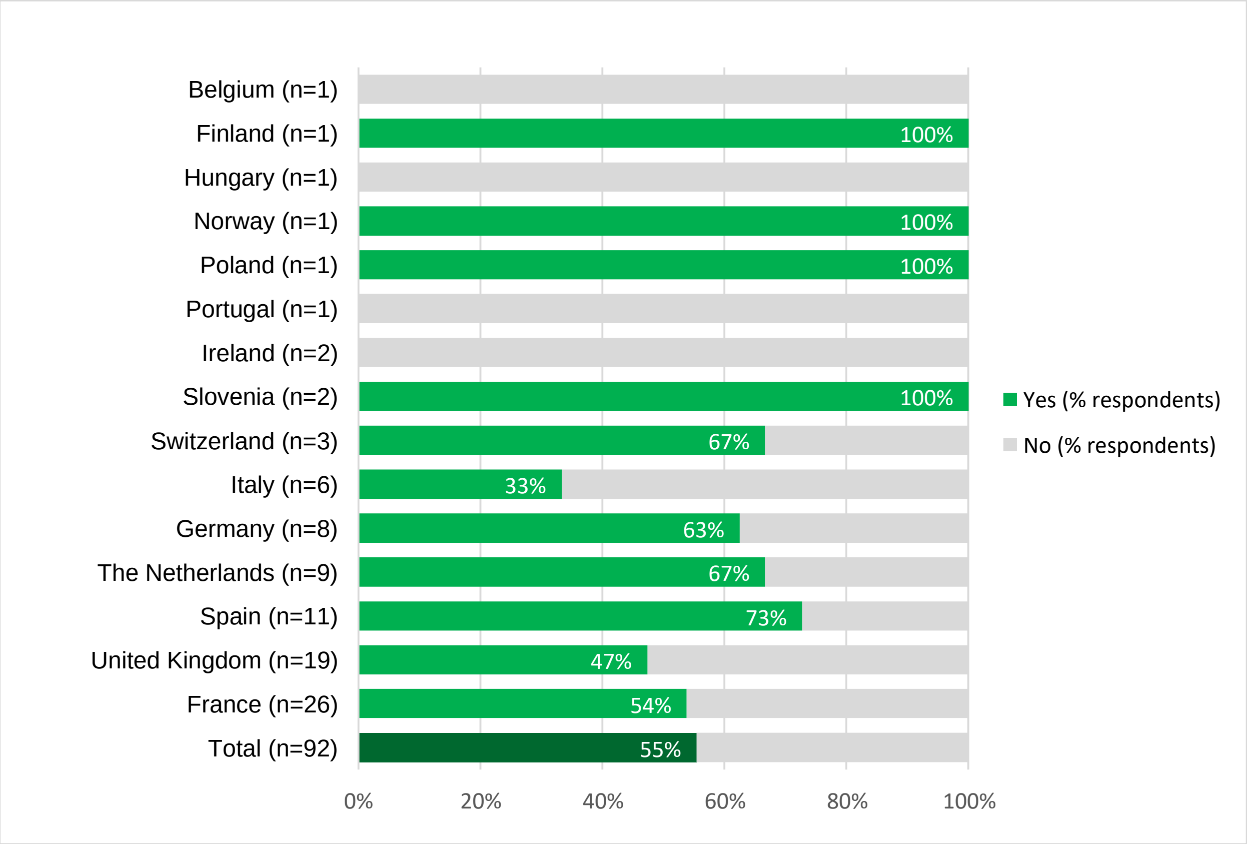

Figure S1: Proportion of respondents who evaluate platelet transfusion recovery via laboratory testing, presented by country of practice, reported in response to survey on European clinical management of Glanzmann's Thrombasthenia.

2. Survey questions

Introduction

The EAHAD Glanzmann's Thrombasthenia Working Group (GTWG) has recently been formed and is pleased to announce their first project, the European Initiative on Glanzmann's Thrombasthenia* to further understand the diagnosis, treatment, unmet needs and overall management of this patient population. This Initiative is funded by an educational grant from Novo Nordisk and aims to raise awareness of this rare disorder, as well as related conditions, with the ultimate aim of improving patient care.

The GTWG invites you to contribute to this initiative by completing this survey, which will ask about your experience of GT management in your clinical practice. We would welcome responses from all physicians across Europe, who have managed or are currently managing patients with GT.

This survey consists of questions that are easy to respond to from memory, without consulting clinical practice records.

Your responses to this survey will help to generate a valuable snapshot of how this rare and debilitating bleeding disorder is currently diagnosed and managed in clinical practice, which is also planned for publication following statistical analysis.

Thank you very much for supporting this valuable initiative,

The EAHAD Glanzmann's Thrombasthenia Working Group, including the project steering committee, Roseline d'Oiron, Mathieu Fiore, Andrea Artoni, Robert Klamroth, Mary Mathias, and Roger Schutgens.

*Glanzmann's Thrombasthenia (GT) is a rare autosomal recessive disorder, characterised by spontaneous and trauma-related mucocutaneous bleeding, with variable expression ranging from easy bruising to fatal haemorrhages. Despite several reports on management of patients with GT, there is currently no European consensus.

Guidance for completing the survey

Please consider only the treatment and management of Glanzmann's Thrombasthenia specifically, not other bleeding disorders. Kindly answer every question to ensure a complete and comparable set of data. The survey will take approx. 15-20 minutes to fill out. You can suspend and continue completing the survey at any time until submission in the end. This may not work on mobile devices.

This survey will cover:

- General information
- Laboratory testing for diagnosis of the disease
- Platelet and red blood cells administration
- Anti-HLA immunisation
- Anti-αIIbβ3 immunisation

- rFVIIa
- Other treatments
- General comments

Personal Information

1. Medical institution

Your affiliation is requested to understand the specialties and the geographic distribution of survey respondents and will not be disclosed for any purpose nor associated with any data presented.

Please provide:

- **Specialty/Department** *(Text box)*
- **Institution** *(Text box)*
- **City** *(Text box)*
- **Country** *(Text box)*

General Information

2. On average, how many patients with Glanzmann's Thrombasthenia (GT) do you follow in your institution?

- <5
- 5–10
- 10–20
- 20–30
- >30

3. What age group(s) of patients with GT do you manage?

- Adults
- Children
- Both

4. Do you usually deliver an emergency card to patients?

- Yes
- No

5. Do you include your patients in a national database?

(Please check all that apply.)

- Yes, in a specific database
- Yes, in a database common to other congenital bleeding disorders
- No
- Other *(please specify)*

6. What frequency of follow-up by the specialised centre do you usually recommend in the long term, apart from any intercurrent event?

- Twice per year
- Once per year
- Every 2 years
- >2 years
- As necessary

7. Which tools are you aware of/do you use in clinical practice to document disease burden in GT patients? Please consider this in the context of a prospective registry trial.

(For each tool, please select from the following options: I know and use this tool; I know but don't use this tool; I don't know this tool.)

- ISTH-BAT
- ITP-BAT
- IWG-ITP surgery BAT
- Euro-QoL-5D
- PedsQL 4.0
- PBAC
- MMAS
- CIS-8
- Other *(please specify)*

Laboratory Testing for Diagnosis of the Disease

8. How do you diagnose GT patients?

(Please check 'Yes', 'No' or 'Not available' for each method.)

- Light Transmission Aggregometry

- Flow cytometry
- Western Blot
- Genetic analysis (ITGA2B and/or ITGB3 sequencing)
- Other *(please specify)*

9. Do you have access to emergency laboratory tests to diagnose GT in your centre?

- Yes
- No

Platelet and Red Blood Cells Administration

10. Are you able to access Human Leukocyte Antigens (HLA)-matched platelets from your local blood bank?

- Yes, without limitations
- Yes, with limitations
- No

11. Have there been occasions in your clinical practice where you would have preferred to use HLA-matched platelets, but were unable to wait for or access them?

- Yes
- No

12. Should all types of platelet concentrates be available to you, which type of platelet products would you usually prefer to use, when time allows?

- Apheresis platelet concentrates
- Apheresis platelet concentrates (HLA identical)
- Pooled random donor platelet concentrates
- No preference

13. If you use HLA-matched platelet concentrates, when do you use them?

- Systematically for all patients
- Never
- Only in specific indications

HLA-Matched Platelet Concentrates

14. Please specify the indications in which you would use HLA-matched platelet concentrates:

(Please check all that apply.)

- In young women of childbearing age even if she is not anti-HLA immunised
- In case of current presence of anti-HLA antibodies, regardless of current or past refractoriness status
- In case of current presence of anti-HLA antibodies, with current or past platelet transfusion refractoriness
- In case of history of anti-HLA antibodies, but without current anti-HLA immunisation, regardless of current or past refractoriness status
- In case of documented current platelet transfusion refractoriness without proven past or current anti-HLA immunisation
- In case of history of platelet transfusion refractoriness without proven past or current anti-HLA immunisation
- Other *(please specify)*

15. Do you evaluate platelet transfusion recovery with laboratory testing?

- Yes
- No

16. If yes, which kind of tests do you use when it is possible?

(Please check all that apply.)

- Platelet count
- Flow cytometry (percentage evaluation of CD41- or CD61-positive transfused platelets)
- Flow cytometry (platelet function testing using PAC-1 or other markers)
- Platelet aggregation testing
- Platelet Function Analyzer (PFA)
- Thromboelastography (-metry)
- Other *(please specify)*

17. How do you define platelet transfusion inefficacy?

(Please check all that apply.)

- Insufficient platelet count increments
- Insufficient proportion of GPαIIbβ3-positive platelets after transfusion by flow cytometry
- Clinical inefficacy despite sufficient amount of platelets (persistent bleeding, absence of prevention of pre/post-operative bleeding, decrease of Hb levels)
- Other *(please specify)*

18. Do you ever use washed red blood cell concentrates (removal of residual platelets) to prevent the development or reactivation of anti-αIIbβ3 immunisation?

- Yes
- No

19. When treating paediatric patients, have you needed to place central venous access devices due to the frequency of platelet transfusions?

- Yes
- No

20. Have you ever given regular prophylactic platelet transfusions in an attempt to reduce bleeding episodes and emergency treatment?

- Yes
- No

Cases of Major Surgery (e.g., total hip replacement)

21. In case of major surgery, how many units of platelets do you generally transfuse pre-operatively?

(Please consider adult patients only.)

- 1
- 2
- >2

22. In case of major surgery, do you perform a second transfusion episode postoperatively?

(Please consider adult patients only.)

- No
- Yes, approximately 12 hours after the first transfusion, systematically

- Yes, approximately 24 hours after the first transfusion, systematically
- Yes, based on transfusion recovery (monitoring via laboratory testing)
- Yes, other *(please specify)*

23. In case of major surgery, if you do perform a second platelet transfusion, how many units would this transfusion include?

(Please consider adult patients only.)

- I don't perform a second platelet transfusion
- 1 unit
- 2 units
- >2 units

24. In case of major surgery, how many days of platelet transfusions would you propose in cases of favourable response (no excessive bleeding)?

(Please consider adult patients only.)

- 1 day (including pre-operative transfusions)
- 2 days
- 3 days
- 4 days
- 5 days
- >5 days

Cases of Minor Surgery (e.g., wisdom teeth extraction)

25. In case of minor surgery, how many units of platelets do you generally transfuse pre-operatively?

(Please consider adult patients only.)

- 1 unit
- 2 units

26. In case of minor surgery, do you perform a second transfusion episode postoperatively?

(Please consider adult patients only.)

- No

- Yes, approximately 12 hours after the first transfusion, systematically
- Yes, approximately 24 hours after the first transfusion, systematically
- Yes, based on transfusion recovery (monitoring via laboratory testing)
- Yes, other *(please specify)*

27. In case of minor surgery, if you do perform a second platelet transfusion, how many units would this transfusion include?

(Please consider adult patients only.)

- I don't perform a second platelet transfusion
- 1 unit
- 2 units

28. In case of minor surgery, how many days of platelet transfusions would you propose in cases of favourable response (no excessive bleeding)?

(Please consider adult patients only.)

- 1 day (including pre-operative transfusions)
- 2 days
- 3 days
- >3 days

Anti-HLA Immunisation

Please consider HLA (Human Leukocyte Antigens) antibodies only when answering questions in this section; anti-αIIbβ3 antibodies will be covered in a subsequent section.

29. Do you perform HLA class-I phenotyping and/or genotyping in GT patients?

- Yes, systematically
- Yes, occasionally
- No

30. Do you check for HLA class-I antibodies?

- Yes
- No

31. If yes in one of the above questions, for what purpose?

(Please check all that apply.)

- To prevent anti-HLA immunisation formation by using HLA-matched platelet concentrates even if the patient has never had anti-HLA antibodies
- To deliver HLA-matched platelet concentrates if possible and necessary in case of anti-HLA immunisation development (with or without platelet transfusion refractoriness)
- To deliver HLA-matched platelet concentrates in case of platelet transfusion refractoriness, but without detected anti-HLA antibodies

32. Do you think that HLA class-I antibodies represent a clinical problem in GT management?

- Yes
- Sometimes
- Rarely
- No

33. Do you follow-up anti-HLA antibodies if they are positive?

- Yes
- No

Anti-αIIbβ3 Immunisation

34. Do you think that anti-αIIbβ3 antibodies represent a clinical problem in GT management?

- Yes
- Sometimes
- Rarely
- No

35. Do you regularly screen for anti-αIIbβ3 antibodies?

- Yes
- No

36. In what situations do you screen anti-αIIbβ3 antibodies?

(Please check all that apply.)

- Before any platelet concentrates administration
- Before any red blood cells administration
- During weeks or months after any platelet concentrates administration
- During weeks or months after any red blood cells administration
- In case of pregnancy
- Never

37. Which of these test(s) do you use to detect anti-αIIbβ3 antibodies?

(Please check all that apply.)

- Indirect MAIPA
- ELISA
- Flow cytometry
- Luminex technology
- Other *(please specify)*

38. Do you evaluate the in vitro effect of the anti-αIIbβ3 isoantibodies on platelet function of normal donor platelets?

- Yes
- No

39. If yes, which of these tests do you use?

- Platelet aggregation testing
- Flow cytometry
- Other *(please specify)*

40. Which of the following risk factors for anti-αIIbβ3 antibodies formation would influence your clinical strategy?

(Response options include: Never, Unlikely, Likely, Always, N/A for each factor)

- Percentage of αIIbβ3 expression or GT type
- Type of pathogenic molecular variants
- Familial history of anti-αIIbβ3 antibodies
- Polymorphisms in immune system genes

- Number of platelet concentrates
- Type of platelet concentrates

rFVIIa

41. Do you consider using rFVIIa despite a lack of antiplatelet immunisation or presence of platelet transfusion inefficacy?

- Never
- Rarely
- Often
- Always

42. Do you consider the site of bleeding (e.g., intracranial haemorrhage) and/or the severity of bleeding (e.g., life-threatening episodes) when selecting rFVIIa rather than platelets?

- No
- Yes

43. Do you consider age and sex (for example, women with child-bearing potential) when using rFVIIa?

- Yes, age
- Yes, sex
- Yes, both
- Neither

44. The recommended dose for the treatment of bleeding episodes and for the prevention of bleeding in patients undergoing surgery or invasive procedures is 90 µg (80–120 µg) rFVIIa per kg body weight every 2–3 hours. A minimum of three doses should be administered to ensure effective haemostasis. Does this dosage reflect your practice?

- Yes
- No

45. On what basis do you adjust this dosage?

(Please check all that apply.)

- Age
- The location of the bleed or the type of surgery
- Efficacy observed on a previous episode
- Response to treatment of the current bleeding episode
- In case of thromboembolic risk factors
- Other *(please specify)*

46. Do you use higher dose of rFVIIa in paediatric patients to alleviate risk of bleeding events?

- Yes
- No
- I only manage adult patients

47. Do you sometimes use a unique dose of 270 µg of rFVIIa to treat minor bleeding episodes?

- Yes
- No

48. In case of major surgery (e.g., total hip replacement), what is the average duration that you would propose covering with rFVIIa in case of favourable response (no excessive bleeding)?

- 1 day
- 2 days
- 3 days
- 4 days
- 5 days
- >5 days

49. In case of minor surgery (e.g., wisdom teeth extraction), what is the average duration that you would propose covering with rFVIIa in case of favourable response (no excessive bleeding)?

- 1 day
- 2 days
- 3 days

- >3 days

50. Have you ever used rFVIIa concomitantly or sequentially with platelet transfusions?

- Yes
- No

Other Treatments

51. Do you use tranexamic acid in the context of surgery?

- Yes
- Yes, only for major surgery
- No

52. Do you use pharmacological thromboprophylaxis in a surgical setting?

- Yes
- No

53. Do you use desmopressin in the setting of GT?

- Often
- Sometimes
- Rarely
- Never

54. Do you prescribe chronic oral iron supplementation?

- Yes
- No

55. Do your patients require intravenous iron supplementation?

- Yes, many
- Yes, some
- No

56. If yes, in which situation?

(Please check all that apply.)

- Digestive intolerance of oral iron
- Inefficacy of oral administration
- Acute anaemia
- Chronic and persistent anaemia

57. Have any of your patients undergone bone marrow transplantation?

- Yes
- No

Future Activities

58. Do you have any further comments regarding the topics covered in this survey and unmet needs in the management of Glanzmann's Thrombasthenia?

(Open text response)

While this survey covered general aspects of the clinical management of Glanzmann's Thrombasthenia, the EAHAD GTWG would also appreciate it if you would be willing to participate in a survey on specific GT management issues, such as those listed below:

- Mouth bleeding
- Nose bleeding
- GI bleeding
- Pregnancy
- Heavy menstrual bleeding
- Use of anticoagulants

59. May we contact you via email to invite you to participate in such a survey?

- No
- Yes, my email address is: *(Text box)*
